# Suprachoroidal hemorrhage associated with pars plana vitrectomy

**DOI:** 10.1186/s12886-021-02062-7

**Published:** 2021-08-11

**Authors:** Bin Mo, Song-Feng Li, Yi Liu, Jun Zhou, Shao-Li Wang, Xiang-Yu Shi

**Affiliations:** Beijing Tongren Hospital, Beijing Ophthalmology & Visual Sciences Key Laboratory, Beijing Tongren Eye Center, Capital Medical University, No. 1 Dong Jiao Min Xiang, Eastern District, 100730 Beijing, China

**Keywords:** Suprachoroidal hemorrhage, Pars plana vitrectomy, Vitrectomized eye

## Abstract

**Purpose:**

To analyze the characteristics, related risk factors, and prognosis of suprachoroidal hemorrhage (SCH) associated with pars plana vitrectomy (PPV).

**Methods:**

Cases of SCH associated with PPV excluding trauma were retrospectively analyzed in Beijing Tongren Hospital between January 2010 and June 2020. The data collected included general data, myopia status, axial length, state of the crystalline lens, SCH onset time, range, treatment method, visual prognosis, and methods of operation and anesthesia. Patients were divided into those with SCH related to the first PPV (Group 1), and SCH related to second intraocular surgery in the vitrectomized eye (Group 2). Patients were also classified by the SCH onset time into either the expulsive suprachoroidal hemorrhage group (ESCH) and the delayed suprachoroidal hemorrhage group (DSCH). The general data, related risk factors, and the visual prognosis of SCH in the different groups were analyzed.

**Results:**

SCH associated with PPV was studied in 28 cases with an incidence of 0.06 %; 16 males and 12 females. The mean age of the patients was (53.51 ± 10.21) years old, the mean follow-up time was (24.94 ± 14.60) days, and the mean axial length was (28.21 ± 3.14) mm. Of these cases, 21 were classified as high myopia, 25 as aphakia/ pseudophakic, and 7 as focal hemorrhage. Silicone oil removal occurred in 12 cases (43 %). Patients in Group 2 were younger than Group 1 (*P* = 0.005). In terms of treatment and prognosis, 5 eyes were simply closely observed, 4 were given single suprachoroidal drainage, 15 were given suprachoroidal drainage combined with silicone tamponade, 2 underwent anterior chamber puncture, and 2 gave up treatment. A follow-up vision: NLP ~ 20/30; among them, 2 eyes with NLP (7.14 %), 6 of ≥ 20/200 (21.43 %). The final outcomes presented a significantly positive correlation with baseline vision but no significant correlation with age or axial length.

**Conclusions:**

SCH has a higher incidence rate after a second intraocular surgery in a vitrectomized eye which is associated with the lack of vitreous support and easier fluctuation of intraocular pressure. SCH associated with PPV is more localized and has a relatively good prognosis; high myopia and aphakic/ pseudophakic eyes are risk factors. Active treatment can effectively improve visual prognosis.

**Trial registration:**

Retrospective case series study, not applicable.

## Background

Suprachoroidal hemorrhage (SCH) is a rare but serious threat to vision, and is one of the most severe complications during or after intraocular surgery [1]. According to onset time, SCH can be divided into intraoperative expulsive suprachoroidal hemorrhage (ESCH) and post-operative delayed suprachoroidal hemorrhage (DSCH) [2]. According to the hemorrhage range, SCH can be divided into focal or diffuse SCH [3]. Blood in the suprachoroidal space can infiltrate the inferior retina, vitreous body, and anterior chambers, causing severe damage to the functioning of ocular tissue, retinal proliferation, and tractional retinal detachment, resulting in complete loss of vision. Furthermore, massive SCH for an extended period can cause cyclodialysis and functional loss of the ciliary body, leading to eventual eyeball atrophy [4, 5].

The incidence rate of SCH varies with different procedures, while the related risk factors and prognosis are also slightly different. As previously reported in the literature, the incidence rate of SCH in vitreous and retinal surgeries is 0.09–4.3 % [6–8]. However, there are few reports of SCH associated with pars plana vitrectomy (PPV), or SCH related to second intraocular surgery in vitrectomized eyes. Herein we have summarized and analyzed cases of SCH associated with PPV from the last 10 years, including the related risk factors and prognosis.

## Methods

### Patients

We retrospectively analyzed 48 654 patients with PPV treated in Beijing Tongren Hospital between January 2010 and June 2020. Of these, 28 cases were associated with SCH. Patients with eye trauma-related SCH were excluded. This study complied with the guidelines of the Declaration of Helsinki.

General data were collected, including sex, age, full medical history, anticoagulant use, condition of the eyes including past surgery, presence of high myopia, axial length, state of the crystalline lens, onset time of SCH, hemorrhage range, treatment method, visual prognosis, and surgery-related factors including the methods of operation and anesthesia. In all cases, vision was assessed using a Snellen chart and was converted to the logarithm of the minimum angle of resolution (LogMAR) VA for computational purposes. The following LogMAR cutoffs were used for non-numeric VA [9]: able to count fingers (CF) = 1.7 LogMAR; able to detect hand movement (HM) = 2.0 LogMAR; light perception (LP) = 2.3 LogMAR; no light perception (NLP) = 3.0 LogMAR.

### Methods

Patients were retrospectively analyzed and divided into those who had SCH related to the first PPV (Group 1), or SCH related to the second intraocular surgery in vitrectomized eyes (Group 2). The cases were further divided into an ESCH group and a DSCH group, according to the onset time. The general data of SCH, related risk factors and visual prognosis in the different groups were analyzed.

### Statistical analysis

Data were analyzed using SPSS17.0 software, with measurement data expressed as means ± SD. A chi-squared test was used for the enumeration of the data. Independent-samples student t-test and one-way ANOVA were used for data analysis. Pearson correlation analysis was used to analyze whether there was a correlation between vision outcome after treatment and age, axial length, and baseline vision. Data were considered statistically significant when *P*< 0.05.

## Results

### General patient data

**Fig. 1:**
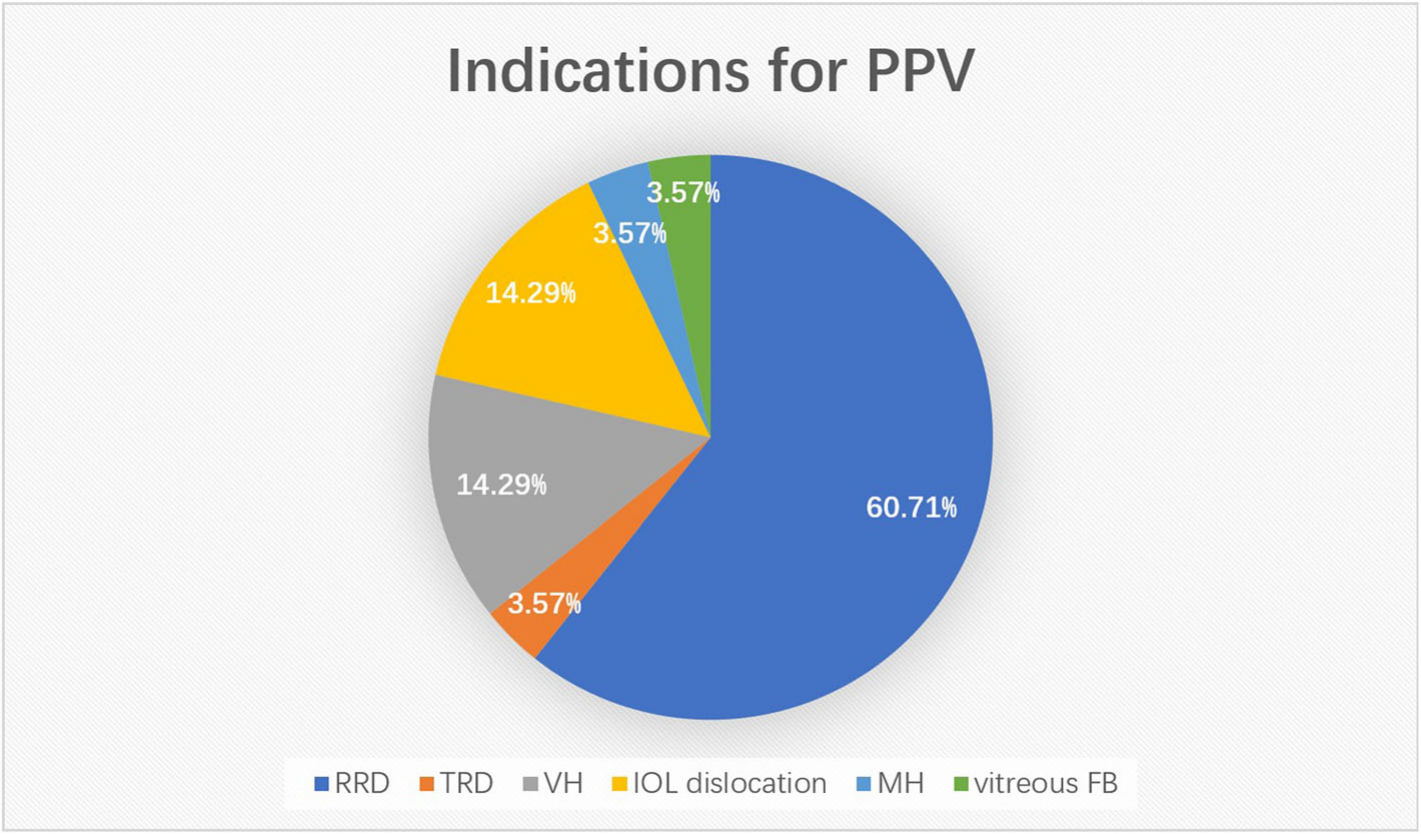
Indications for PPV in this study (figures are percentages). RRD = rhegmatogenous retinal detachment; TRD = tractional retinal detachment; VH = vitreous hemorrhage; MH = macular hole; vitreous FB = vitreous foreign body

Among the 48 654 patients who underwent PPV in our hospital in the past 10 years, 28 patients with SCH were selected (an incidence of 0.06 %). The patients included 16 males and 12 females, with a mean age of (53.51 ± 10.21) years old and a mean follow-up time of (24.94 ± 14.60) days. Among them, 10 cases had other medical conditions, 1 had a medical history of anticoagulant drug use, and 21 had high myopia with a mean axial length of (28.21 ± 3.14) mm. Regarding the state of the crystalline lens, 3 had phakic eyes, 9 aphakic eyes, and 16 pseudophakic eyes (see Table 1 for more details). Indications of first PPV in our study included 17 cases of rhegmatogenous retinal detachment, 1 case of traction retinal detachment, 4 cases of vitreous hemorrhage, 4 cases of IOL dislocation, 1 case of macular hole, and 1 case of a vitreous foreign body ( Fig. 1). Except for 6 cases of cataract and glaucoma after vitrectomy, the other 22 cases were treated with posterior perfusion including 15 of 20G and 7 of 23G. After SCH occurrence, 2 cases were left with perfluorocarbon liquid, 3 patients with silicone oil, and 17 patients with perfusion fluid.

**Table 1:** General data of SCH associated with PPV

Total number of eyes (n)	28
Total number of patients (n)	28
Male: Female	16:12
Mean age (y)	53.51 ± 10.21
Follow-up time (d)	24.94 ± 14.60
Anticoagulant drug use	1: 27
High myopia	21: 7
Mean axial length (mm)	28.21 ± 3.14
State of crystalline lens	
Phakic eyes	3
Aphakic eyes	9
Pseudophakic eyes	16
local anesthesia: general anesthesia	24: 4
ESCH: DSCH	24: 4
Focal SCH: Diffuse SCH	7: 21

### Pathogenesis of PPV-related SCH

**Fig. 2:**
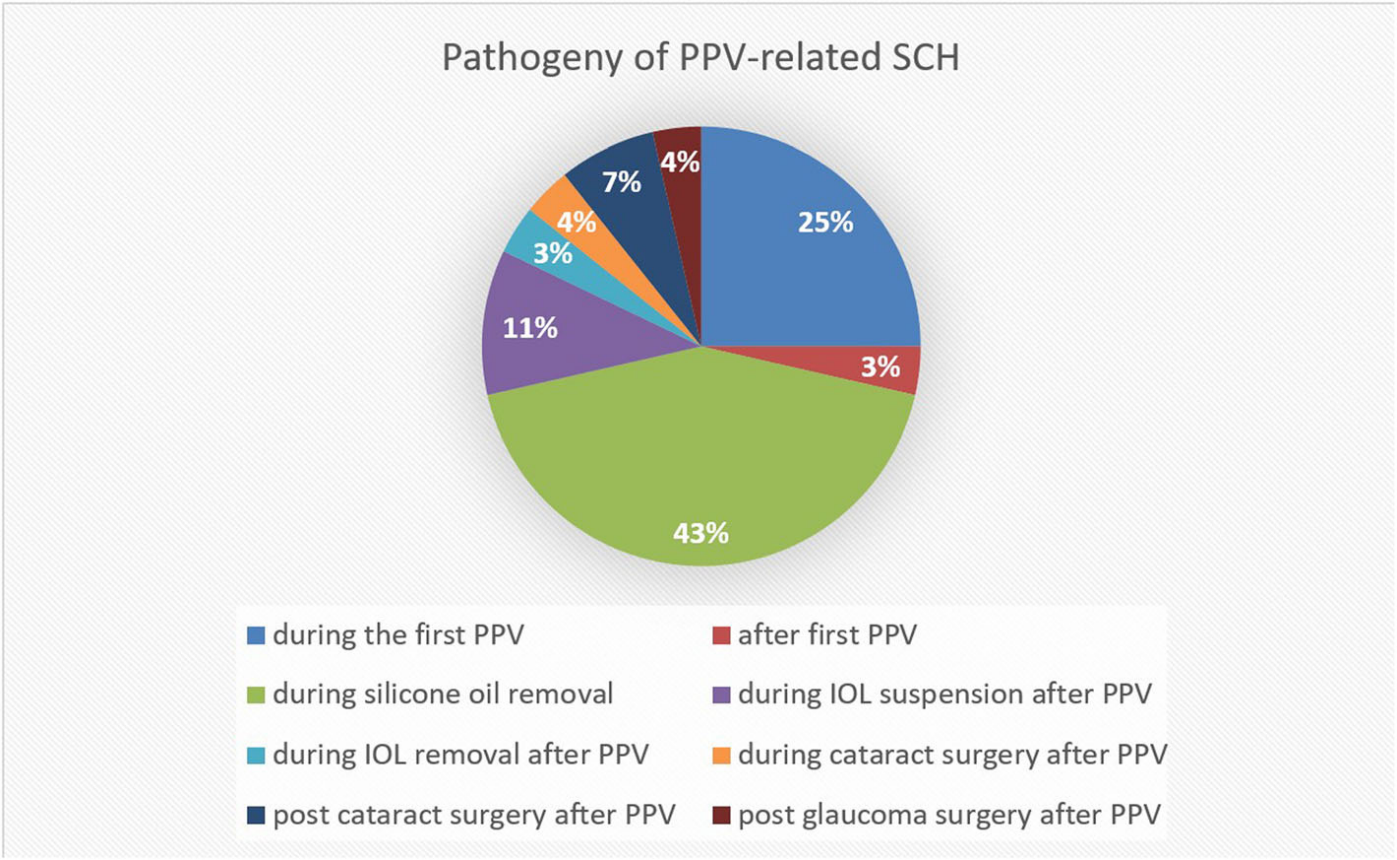
Pathogenesis of SCH associated with PPV

Of the patients studied, 8 showed SCH related to the first PPV, and 20 were related to the second intraocular surgery in vitrectomized eyes (71.43 %). Among them, silicone oil removal resulted in the highest occurrence of SCH, with 12 cases (43 %), 7 of which occurred during the first PPV (25 %), 3 during IOL suspension after PPV, 1 during IOL removal after PPV, 1 during cataract surgery after PPV, 1 after first PPV, 2 in post-cataract surgery after PPV, and 1 in post glaucoma surgery after PPV (see Fig. 2).

### Comparison of SCH between first PPV group and second intraocular surgery in vitrectomized eyes group (see Table 2 for more details)

**Table 2:** Comparison of SCH between the first PPV group and the second intraocular surgery in vitrectomized eyes group

	SCH related to first PPV group (8 cases)	SCH related to second intraocular surgery in vitrectomized eyes group (20 cases)	*P*-value
Male: Female	2: 6	13: 7	0.096
Age (y)	61.88 ± 8.71	50.55 ± 8.90	0.008
Mean axial length (mm)	27.70 ± 3.47	28.09 ± 3.15	0.786
Hemorrhage range (focal: diffuse)	2: 6	5: 15	0.096
Hemoorhage duration (ESCH: DSCH)	7: 1	17: 3	1.000
Baseline VA	1.73 ± 0.59	1.74 ± 0.64	0.968
Final VA	1.48 ± 0.83	1.97 ± 0.74	0.174

Eight patients were included in Group 1, with 2 males and 6 females, with a mean age of (61.88 ± 8.71) years and a mean axial length of (27.70 ± 3.47) mm. Twenty cases were included in Group 2, with 13 males and 7 females, a mean age of (50.55 ± 8.90) years, and a mean axial length of (28.09 ± 3.15) mm. A chi-squared test was applied for sex on both groups, with *P* = 0.096, thus showing no statistical significance. An independent-samples t-test was used for age, with *P* = 0.008, showing statistical significance. The latter was also applied in the analysis of axial length, with *P* = 0.786, showing no statistical significance. The baseline vision was 1.73 ± 0.59 in Group 1 and 1.74 ± 0.64 in Group 2, with *P* = 0.968 using the independent-sample t-test. The final vision was 1.48 ± 0.83 in Group 1 and 1.97 ± 0.74 in Group 2, with *P* = 0.174, showing no statistical significance.

There were 2 cases of focal hemorrhage and 6 cases of diffuse hemorrhage in Group 1, while 5 focal and 15 diffuse hemorrhages occurred in Group 2, with a chi-squared test indicating that there was no statistically significant difference (*P* = 0.096). There were 7 cases of ESCH and 1 of DSCH in Group 1, while 17 ESCH and 3 DSCH occurred in Group 2 with a chi-squared test presenting no statistically significant difference (*P* = 1.00).

### Comparison between ESCH and DSCH (See Table 3 for more details)

**Table 3:** Comparison of ESCH and DSCH

	ESCH group (24 cases)	DSCH (4 cases)	*P*-value
Male: Female	12: 12	3: 1	0.600
Age (y)	54.29 ± 9.65	50.75 ± 13.94	0.654
Mean axial length (mm)	28.10 ± 3.11	27.27 ± 4.04	0.729
Hemorrhage range (focal: diffuse)	6: 18	1: 3	1.000
Baseline VA	1.73 ± 0.55	1.78 ± 1.04	0.927
Final VA	1.85 ± 0.77	1.71 ± 1.00	0.792

With regards to PPV-related cases, the incidence rate of ESCH was significantly greater (24 patients, 85.71 %) than for DSCH (4 patients, 14.29 %). In the ESCH group, 12 were male and 12 were female, with a mean age of (54.29 ± 9.65) years, and a mean axial length of (28.10 ± 3.11) mm. In comparison, the DSCH group included 3 males and 1 female, with a mean age of (50.75 ± 13.94) years and a mean axial length of (27.27 ± 4.04) mm. The influence of sex in both groups was analyzed using the chi-squared test (*P* = 0.60) and age was analyzed by an independent-sample t-test (*P* = 0.654), both of which showed no statistically significant differences. The baseline vision was 1.73 ± 0.55 in the ESCH group and 1.78 ± 1.04 in the DSCH group, and the final vision outcome was 1.85 ± 0.77 in the ESCH group and 1.71 ± 1.00 in the DSCH group. These differences were not significant, as shown by the independent-sample t-test (*P* = 0.927 and *P* = 0.792 for the baseline vision and final vision, respectively).

There were 6 cases of focal and 18 of diffuse hemorrhages in the ESCH group, compared with 1 focal and 3 diffuse hemorrhages in the DSCH group. A chi-squared test indicated that there was no statistically significant difference (*P* = 1.00).

### Analysis of SCH treatment, vision outcome, and vision-related factors

With regards to SCH, 5 patients were simply observed, 4 were given single suprachoroidal drainage, 15 were given suprachoroidal drainage combined with silicone tamponade, 2 underwent anterior chamber puncture, and 2 abandoned treatment. One patient underwent a single suprachoroidal drainage and was injected with rt-PA (recombinant tissue plasminogen activator, rt-PA) in the suprachoroidal space on the fourth day after the occurrence of DSCH and drained after 4 h (see Case 2 for further details). Patients were followed up and the final corrected vision was seen to be: no light perception (NLP) ~ 20/30; among them, 2 eyes showed NLP (7.14 %), 16 could detect hand motions (HM) to light perception (LP), 4 could count fingers (CF) ~ 20/400, and 6 of index ≥ 20/200 (21.43 %) (See Table 4 for details). The final vision was significantly positively correlated with the baseline vision (*r* = 0.545, *P* = 0.000), but not significantly correlated with age (*r*=-0.113, *P* = 0.427) or axial length (*r* = 0.073, *P* = 0.611).

**Table 4:** Analysis of treatment in SCH, vision outcome

Treatment in SCH	Number
Observe	5
Suprachoroidal drainage	4
Suprachoroidal drainage combining silicone tamponade	15
Anterior chamber puncture	2
Abandon	2
Visual outcome	Number
NLP	2(7.14 %)
LP ~ HM	16(57.14 %)
CF ~ 20/400	4(14.28 %)
≧ 20/200	6(21.43 %)

### In-depth analysis of two specific cases of SCH

**Fig. 3:**
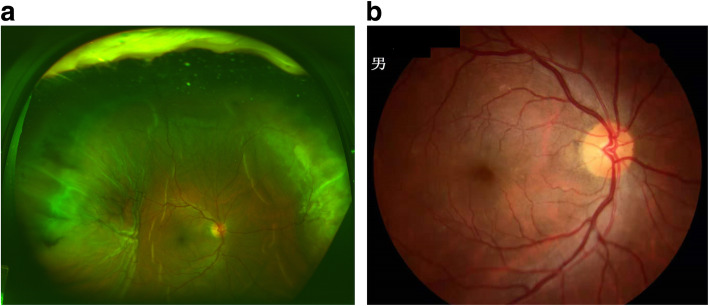
Fundus image of Case 1. **a** is a fundus image of the fifth day of ESCH; **b** is a fundus image of the fourth month of ESCH showing that the retina was flat

Case 1: patient was a 57-year-old male, with a past medical history of hypertension, heart disease, heart stenting, and oral anticoagulant drug treatment (warfarin was stopped five days before surgery and replaced by low-molecular-weight heparin sodium injection). The patient was given PPV combing IOL removal under general anesthesia due to IOL dislocating to the vitreous space. During PPV, ESCH occurred which was found to be diffuse SCH and was observed with medication. After four months, the patient’s vision was 20/30 with a flat fundus (Fig. 3).

**Fig. 4:**
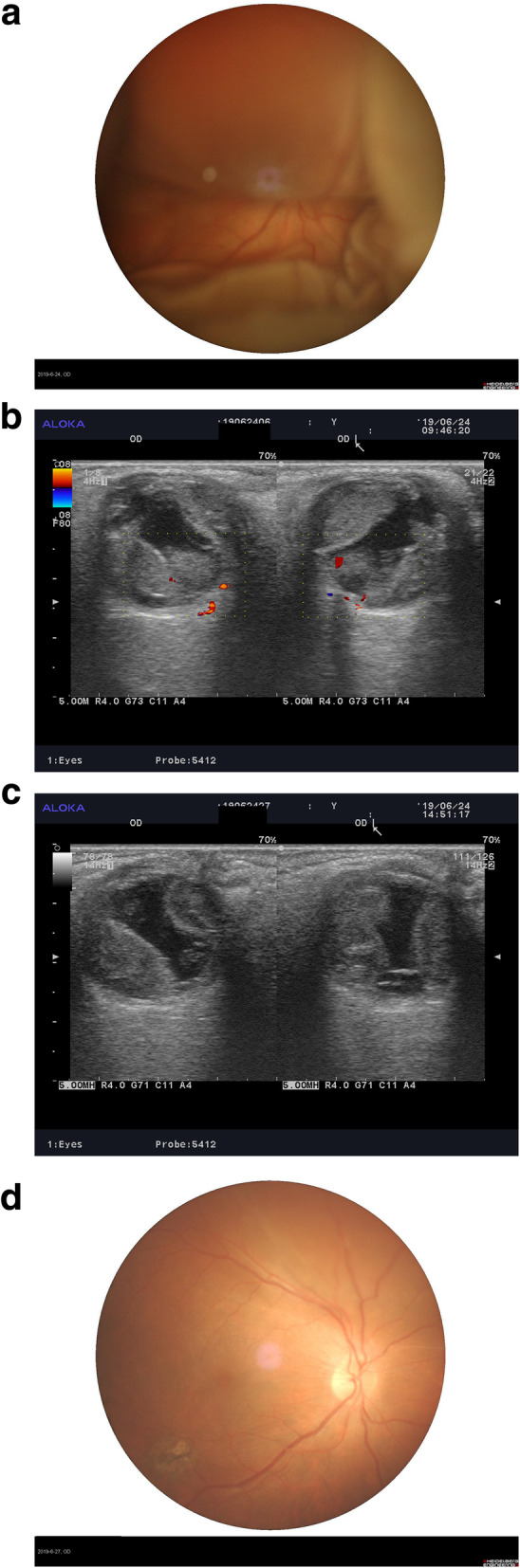
Fundus image and ultrasound examination of Case 2. **a** is a fundus image of the fourth day of DSCH; **b **is an ultrasound examination of the fourth day before rt-PA injection; **c** is an ultrasound examination showed partial liquidation of SCH 4 h after rt-PA injection; **d** is a fundus image of the third day after choroidal drainage showing that a bulge can only be seen in the upper choroid

Case 2: patient was a 46-year-old male who had swelling with pain in the right eye for three hours after the second IOL ciliary sulcus implantation in a vitrectomized eye. The patient had previously received PPV and foreign body removal from the right eye two months before. The vision was LP and the intraocular pressure was 41 mmHg, with diffuse SCH involving the posterior pole with local retinal detachment but no retinal hole. The kissing sign could be seen on ultrasound examination. As no significant change occurred after four days, the patient was given a rt-PA injection in the suprachoroidal space and single suprachoroidal drainage was performed. Three days after drainage, the patient’s vision was 20/60 with an intraocular pressure of 8 mmHg, and a bulge was only seen in the upper choroid. Reexamination was carried out two months after surgery at which time the patient’s vision was 20/30 and both the fundus retina and choroid were flat (Fig. 4).

## Discussion

SCH, defined as the accumulation of blood in the suprachoroidal space between the choroid and sclera, is a rare but severe complication during or after intraocular surgery that seriously threatens vision. The condition causes continuous retinal detachment, secondary glaucoma or hypotony, leading to a prognosis of poor vision, and even vision loss and eyeball atrophy. The mechanism of SCH remains unclear. Previous research has shown that the pathophysiological process of SCH involves multiple factors, and one of the induction factors is a sudden drop in intraocular pressure [1, 4, 5]. The long posterior ciliary artery is particularly fragile where it penetrates from the sclera into the suprachoroidal space and is easily ruptured; excessive low intraocular pressure causes rupture of the long or short posterior ciliary artery, resulting in SCH [1, 4, 5].

According to literature reports, the incidence rate of SCH during PPV is 0.09–4.3 % [7, 8], and 0.8 % [8] after PPV. The risk factors of SCH associated with PPV are mainly related to high myopia, aphakic/ pseudophakic eyes, surgical treatment of retinal detachment, and retinal freezing [5, 8, 10, 11]. However, the morphological characteristics and visual prognosis of SCH associated with PPV have not been reported. We summarized cases from our hospital over the last 10 years for cases of SCH related to a first PPV and SCH related to a second intraocular surgery in vitrectomized eyes, in an attempt to analyze the related risk factors, morphological characteristics, and vision prognosis.

Among the 48 654 patients who underwent PPV in our hospital in the past 10 years, 28 SCH patients were selected (an incidence of 0.06 %), with 8 cases of SCH related to thefirst PPV and 20 related to the second intraocular surgery in vitrectomized eyes (71.43 %). Cases involving silicone oil removal surgery were the most abundant (12 cases; 43 %). The incidence rate of SCH related to second intraocular surgery in vitrectomized eyes was higher than that occurring in the first PPV surgery. We consider that in the second surgery in vitrectomized eyes, the frequent fluctuation of intraocular pressure occurred without the vitreous body, particularly due to repeated fluid-air exchange in the silicone oil removal surgery. With regards to the age at onset, the group comprising second intraocular surgery in vitrectomized eyes were slightly younger than the first PPV group. In our opinion, eye factors such as a repeated fluctuation of intraoperative intraocular pressure, high myopia, and aphakic/pseudophakic eyes were more important factors. The incidence rate of focal suprachoroidal hemorrhage associated with PPV was 25 %, and no incidence rate regarding focal suprachoroidal hemorrhage has been reported in the literature.

There were 24 cases of ESCH (85.7 %), which was significantly higher than the DSCH incidence. Recent studies have shown the incidence rate of DSCH after PPV to be 0.8 %, which is similar to the rate of 1 % during surgery [9, 12]. However, the incidence rate of DSCH was lower in our study; this might be associated with the loss of patients who did not visit our hospital or received treatment in the outpatient department. Additionally, of the four DSCH cases, one occurred after vomiting while the induction factors in the other three were undetermined. Literature reports have demonstrated that SCH has significant correlations with advanced age, long axial length, rhegmatogenous retinal detachment, extensive retinal photocoagulation, oral anticoagulant drug use, and postoperative vomiting [8, 12–14]. In our study, there was high myopia in 75 % of cases, a mean axial length of 28.21 ± 3.14 mm, and aphakic and pseudophakic eyes in 89.29 % of cases, a mean age of 53.51 ± 10.21 years old, and only one case of oral anticoagulant use. Therefore, long axial length and aphakic/ pseudophakic eyes are absolute risk factors for SCH and are associated with the increased fragility of blood vessels in high myopia and choroidal effusion that is easily caused by a large vitreous space and little support in the sclera and vitreous body. For the prevention of SCH, we suggest that intraocular pressure fluctuation should be heeded during the operation, especially in the process of silicone oil removal. Every time the instrument is replaced, a scleral nail should be used to plug the puncture opening to seal the incision; the intraoperative instrument should be gently pressed against the eyeball to reduce the possibility of SCH occurrence.

In the 28 cases of SCH associated with PPV, 5 eyes were simply observed, 4 were given single suprachoroidal drainage, 15 were given suprachoroidal drainage combined with silicone tamponade, 2 underwent anterior chamber puncture, and 2 abandoned treatment. According to literature reports, for non-traumatic surgery-related SCH, 70 % of patients’ vision was less than 20/400, with 12–57 % of SCH cases presenting NLP, which was even as high as 86 % in the end [4]. Among our cases, 7.14 % presented NLP and 21.43 % ≥ 20/200. The prognosis of SCH associated with PPV was relatively good. We suggest the following possible reasons:: (1) In normal vitreous eyes, when SCH occurs, the high bulge of the choroid would forcibly squeeze out the vitreous, causing the iris and crystalline lenses to shift forwards. The fronted vitreous and ocular contents would cause traction in the retina and choroid, resulting in further expansion of the SCH and retinal detachment, whereas in vitrectomized eyes, without strong traction of the vitreous, SCH tends to be localized and retinal detachment is rare. (2) Since there is no vitreous in vitrectomized eyes, the possibility of post-operative retinal proliferation is greatly reduced, thus decreasing the occurrence of continuous retinal detachment. (3) SCH occurs in vitrectomized eyes, and the postoperative inflammatory response is usually mild; it also reduces the postoperative proliferation. (4) There is internal perfusion during PPV, which can be rapidly increased when SCH occurs, thus, ocular hypotension usually does not last for an extended period. (5) The rise of postoperative intraocular pressure is controllable because “non-vitreous fluid” can easily be discharged from the anterior chamber angle and there is no extended severe pupillary block [15]. (6) The follow-up time is short and the assessment of long-term vision would increase the validity of the findings.

Apart from a correlation with baseline vision, the visual prognosis of SCH is also related to the form of choroidal hemorrhage. Localized SCH or SCH without macular involvement has a better prognosis. In the case of diffuse SCH, the question of how to improve the prognosis as much as possible remains. The currently accepted treatment option is to perform suprachoroidal drainage surgery approximately two weeks after the occurrence of hemorrhage since the liquefaction of the blood clot at this time benefits the drainage of SCH [16]. However, even when the choroid has been reattached, it is impossible for the ocular tissue function to recover from damage caused by long-term hemorrhage. Therefore, early liquefaction and removal of blood clots in the suprachoroidal space to minimize the damage has become the core treatment in SCH, which is possible with the application of t-PA. In 1998, Kwon et al. first injected t-PA into the suprachoroidal space to treat SCH in animal experiments, and subsequently, there have been cases reported since 2012 [17–21]. In our study, one patient developed DSCH after a second IOL implantation in vitrectomized eyes (Case 2 above). On the fourth day after SCH, rt-PA (Alteplase, 10 µg/0.1 mL) was injected into the suprachoroidal space, where drainage was performed afterwards. This improved the patient’s vision from LP to 20/30 which significantly improved the prognosis. Therefore, for diffuse SCH, we need to use aggressive treatment to further improve the prognosis.

There are, however, a number of shortcomings in this study: (1) The effects of intraoperative surgery were not counted, such as intraocular photocoagulation and retinal freezing; (2) The follow-up time was short and long-term visual prognosis was not analyzed; (3) The treatment methods such as t-PA treatment were not summarized because the SCH case numbers were low. Therefore, sample numbers should be increased and a multicentric study should be performed to reach a definite conclusion.

In conclusion, the incidence rate of SCH associated with second intraocular surgery in vitrectomized eyes is higher which is related to the lack of vitreous support and is caused by easier fluctuation of intraocular pressure. SCH associated with PPV is more likely to be localized and has a relatively good prognosis. High myopia and aphakic/pseudophakic eyes remain the highest risk factors for SCH. Adequate preoperative assessment of the patient’s general condition should be performed, and intraoperative surgery should be careful to avoid fluctuation in intraocular pressure, especially for patients undergoing second intraocular surgery in vitrectomized eyes. Furthermore, attention should be paid to risk prevention; the incision closure should be made carefully in order to reduce the risk of wound leakage, and any side-effects of anesthesia should be treated promptly and aggressively once it occurs to save the patient’s vision.

## Data Availability

The datasets used and/or analysed during the current study are available from the corresponding author on reasonable request.

## Abbreviations

SCH: Suprachoroidal hemorrhage
ESCH: Expulsive suprachoroidal hemorrhage
DSCH: Delayed suprachoroidal hemorrhage
PPV: Pars plana vitrectomy
LogMAR: Logarithm of the minimum angle of resolution
rt-PA: Recombinant tissue plasminogen activator

## References

[1] Moshfeghi DM, Kim BY, Kaiser PK, Sears JE, Smith SD (2004). Appositional suprachoroidal hemorrhage: a case-control study. Am J Ophthalmol.

[2] Tuli SS, WuDunn D, Ciulla TA, Cantor LB (2001). Delayed suprachoroidal hemorrhage after glaucoma filtration procedures. Ophthalmology.

[3] Reynolds MG, Haimovici R, Flynn HW, Dibernardo C, Byrne SF, Feuer W (1993). Suprachoroidal hemorrhage: clinical features and results of secondary surgical management. Ophthalmology.

[4] Wang LC, Yang CM, Yang CH, Huang JS, Ho TZ, Lin CP, Chen MS (2008). Clinical characteristics and visual outcome of non-traumatic suprachoroidal haemorrhage in Taiwan. Acta Ophthalmol.

[5] Sukpen I, Stewart JM. Acute intraoperative suprachoroidal hemorrhage during small-gauge pars plana vitrectomy. Retinal Cases Brief Rep. 2018;12:suppl1,S9–11. 10.1097/ICB.0000000000000659 29155697

[6] Ishida M, Takeuchi S (2000). Vitrectomy for the treatment of expulsive hemorrhage. Jpn J Ophthalmol.

[7] Machemer R, Laqua H (1978). A logical approach to the treatment of massive periretinal proliferation. Ophthalmology.

[8] Reibaldi M, Longo A, Romano MR, Cennamo G, Mariotti C, Boscia F, Bonfiglio V, Avitabile T (2015). Delayed suprachoroidal hemorrhage after pars plana vitrectomy: five-year results of a retrospective multicenter cohort study. Am J Ophthalmol.

[9] Ch’ng SW, Patton N, Ahmed M, Ivanova T, Baumann C, Charles S, Jalil A (2018). The Manchester Large Macular Hole Study: Is it time to reclassify large macular holes?. Am J Ophthalmol.

[10] Tabandeh H, Sullivan PM, Smahliuk P, Flynn HW, Schiffman J (1999). Suprachoroidal hemorrhage during pars plana vitrectomy risk factors and outcomes. Ophthalmology.

[11] Iwama Y, Nakashima H, Emi K, Bando H, Ikeda T (2018). Influence of surgical procedures and instruments on the incidence of suprachoroidal hemorrhage during 25-gauge pars plana vitrectomy. Ophthalmol Retina.

[12] Jin W, Xing Y, Xu Y, Wang W, Yang A (2014). Management of delayed suprachoroidal haemorrhage after intraocular surgery and trauma. Grafes Arch Clin Exp Ophthalmol.

[13] Chandra A, Xing W, Kadhim MR, Williamson TH (2014). Suprachoroidal hemorrhage in pars plana vitrectomy: risk factors and outcomes over 10 years. Ophthalmology.

[14] Tabandeh H, Flynn HW (2001). Suprachoroidal hemorrhage during pars plana vitrectomy. Curr Opin Ophthalmol.

[15] Huang Y, Liao Q, Yuan R (2015). Management and visual outcome of SCH in Vitrectomized eye. Med Hypotheses.

[16] Vuković D, Petrović S, Paović P (2015). Secondary surgical management of suprachoroidal hemorrhage during pars plana vitrectomy. Vojnosanit Pregl.

[17] Kwon OW, Kang SJ, Lee JB, Lee SC, Yoon YD, Oh JH (1998). Treatment of suprachoroidal hemorrhage with tissue plasminogen activator. Ophthalmologica.

[18] Fei P, Jin HY, Zhang Q, Li X, Zhao PQ (2018). Tissue plasminogen activator-assisted vitrectomy in the early treatment of acute massive suprachoroidal hemorrhage complicating cataract surgery. Int J Ophthalmol.

[19] Kunjukunju N, Gonzales CR, Rodden WS (2011). Recombinant tissue plasminogen activator in the treatment of suprachoroidal hemorrhage. Clin Ophthalmol.

[20] Matsumoto K, Matsumoto CS, Shinoda K, Watanabe E, Mizota A (2012). Tissue plasminogen activator-assisted vitrectomy for ruptured eye with suprachoroidal hemorrhage. Case Rep Ophthalmol.

[21] Murata T, Kikushima W, Imai A, Toriyama Y, Tokimitsu M, Kurokawa T (2011). Tissue-type plasminogen activator-assisted drainage of suprachoroidal hemorrhage showing a kissing configuration. Jpn J Ophthalmol.

